# Effect of Water Content on Ethanol Steam Reforming in the Nonthermal Plasma

**DOI:** 10.1021/acsomega.2c07431

**Published:** 2023-03-07

**Authors:** Bogdan Ulejczyk, Łukasz Nogal, Michał Młotek, Krzysztof Krawczyk

**Affiliations:** † Faculty of Chemistry, Warsaw University of Technology, Noakowskiego 3, 00-664 Warsaw, Poland; ‡ Faculty of Electrical Engineering, Warsaw University of Technology, Pl. Politechniki 1, 00-661 Warsaw, Poland

## Abstract

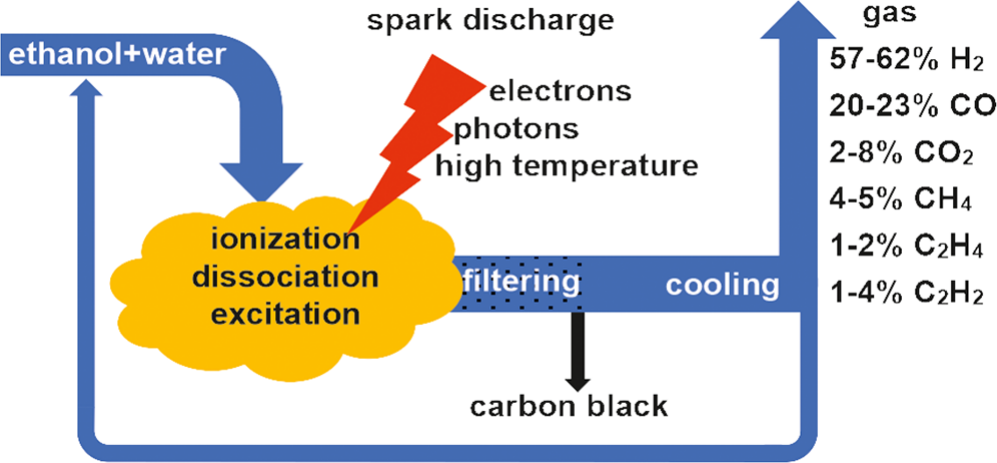

Ethanol steam reforming can be a source of green hydrogen. The process of producing hydrogen from ethanol is very complex. Catalysts designed for this process often become deactivated due to coke deposition. In this work, a plasma reactor was used, which is insensitive to disturbance induced by coke. The research focused on determining the influence of steam on the course of the process. The optimal water/ethanol molar ratio was found to be 4. The energy efficiency was the highest at this ratio, 22.5 mol(H_2_)/kW h. At the same time, a high ethanol conversion (92%) was obtained. It was also observed that the conversion of steam was many times lower than that of ethanol. However, water shortage caused a rapid increase in coke, acetylene, and ethylene production.

## 1 Introduction

Many countries are adopting a strategy of achieving carbon neutrality over several decades. The EU, the US, and Japan plan to accomplish this neutrality in 2050, and China in 2060. One of the paths to decarbonizing the economy is developing a hydrogen economy. Currently, the most important source of hydrogen is natural gas steam reforming, but hydrogen from natural gas is used in the production of ammonia and metal refining. It is not used on a large scale in the power industry. The use of natural gas leaves a carbon footprint on the environment. Therefore, using hydrogen from natural gas for energy production is a temporary solution to reduce coal consumption. Coal has a carbon footprint twice as large as natural gas (Table 1). CH_4_ and C_8_H_18_ are substances representing natural gas and gasoline, respectively.

**Table 1:** Carbon Footprint and Higher Heating Value of Various Species

species	HHV, kW h/mol_species_	CO_2_ emission, mol	carbon footprint, kg(CO_2_)/kW h
CH_4_	0.247	1	0.178
C_8_H_18_	1.519	8	0.232
C	0.109	1	0.403
H_2_	0.079	0	0

Achieving carbon neutrality requires the development of an effective method of obtaining hydrogen from renewable resources, e.g., water or biomass. Water decomposition technologies are expensive. Therefore, much work is devoted to converting biomass to hydrogen. There are different ways to process biomass. One is ethanol fermentation and ethanol steam reforming. The ethanol production technology is very well developed, while the ethanol steam reforming is still problematic. There is a C–C bond in ethanol, that causes the formation of much coke. Coke deposits on catalysts’ surfaces and deactivates them.^1−5^ Therefore, in the process of ethanol reforming, it is worth using plasma reactors, the operation of which is not disturbed by coke. Zhu^6^ and Baránková^7^ generated plasma in liquid substrates. Burlica^8^ and Wang^9^ used reactors with a gliding discharge, where a high-speed gas stream is necessary. Barrier,^10,11^ corona,^12^ microwave,^13^ and spark^14^ discharge reactors were also insensitive to coke.

The resistance to a disturbance in reactor function caused by coke is an excellent advantage of plasma reactors. However, the formation of coke reduces the efficiency of hydrogen formation. Therefore, coke production should be minimized and can be realized using excess water. However, using large amounts of water increases the costs of heating and evaporating the substrates. As a result, hydrogen production can be too expensive.

Theoretically, the production of hydrogen from a mixture of water and ethanol (eq 1) can be very efficient.
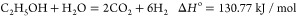
1The productivity and energy efficiency can reach 6 mol(H_2_)/mol(C_2_H_5_OH) and 165 mol(H_2_)/kW h, respectively. However, lower values are achieved in practice due to competitive reactions and energy losses. Generally, higher efficiency of the hydrogen production process is obtained in discharges in which high gas temperatures can be obtained.

Zhu^6^ reported that in a microwave discharge with a power of 900 W, the hydrogen production was 20 mol/h, and the energy yield was 22 mol(H_2_)/kW h. The products were H_2_, CO, CO_2_, CH_4_, C_2_H_2_, C_2_H_4_, C_2_H_6_, CH_3_OH, CH_3_CHO, and coke.

Zhu^12^ reported that in a corona discharge with a power of 15 W, the hydrogen production was 0.15 mol/h, and the energy yield was 10 mol(H_2_)/kW h. The gaseous products were H_2_, CO, CO_2_, CH_4_, and C_2_H_6_. In our previous work,^10^ we reported that in a barrier discharge with a power of 20 W, the hydrogen production was 0.13 mol/h, and the energy yield was 6.15 mol(H_2_)/kW h. The products were H_2_, CO, CO_2_, CH_4_, C_2_H_4_, C_2_H_6_, and coke.

Using a mixture of air and ethanol (eq 2) makes it possible to achieve greater energy efficiency in hydrogen production.
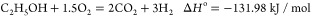
2The productivity can reach 3 mol(H_2_)/mol(C_2_H_5_OH), while the energy yield is not thermodynamically limited because the reaction is exothermic. Guo^15^ reported that in a microwave discharge with a power of 700 W, the energy yield of hydrogen production from a mixture of air and ethanol was 38.5 mol(H_2_)/kW h. However, due to the greater possible productivity, hydrogen production from a mixture of water and ethanol is the most interesting. Because ethanol needs to be produced and its quantity is limited, efforts should be made to obtain as much hydrogen as possible from the ethanol used. Water is readily available and cheap. Therefore, currently, in commercial-scale steam reforming of natural gas, excess water is used to improve the use of natural gas.

This paper presents the effect of water content in the mixture introduced into the reactor on the efficiency of hydrogen production. The water content is an important parameter affecting steam reforming of various substrates. A novelty is a study of ethanol steam reforming in the spark discharge in a wide range of water/ethanol molar ratios. So far, the influence of this parameter on the ethanol steam reforming process in the spark discharge has not been studied. The studies were carried out in a stoichiometric mixture, i.e., a water/ethanol molar ratio equal to 3, with excess water (water/ethanol molar ratio equal to 4, 5, and 6) and water deficiency (water/ethanol molar ratio equal to 2). The aim was to confirm that an increase in the water/ethanol molar ratio reduces coke production. In contrast, a decrease in this ratio increases the energy efficiency of hydrogen respectively.

## 2 Materials and Methods

**Figure 1:**
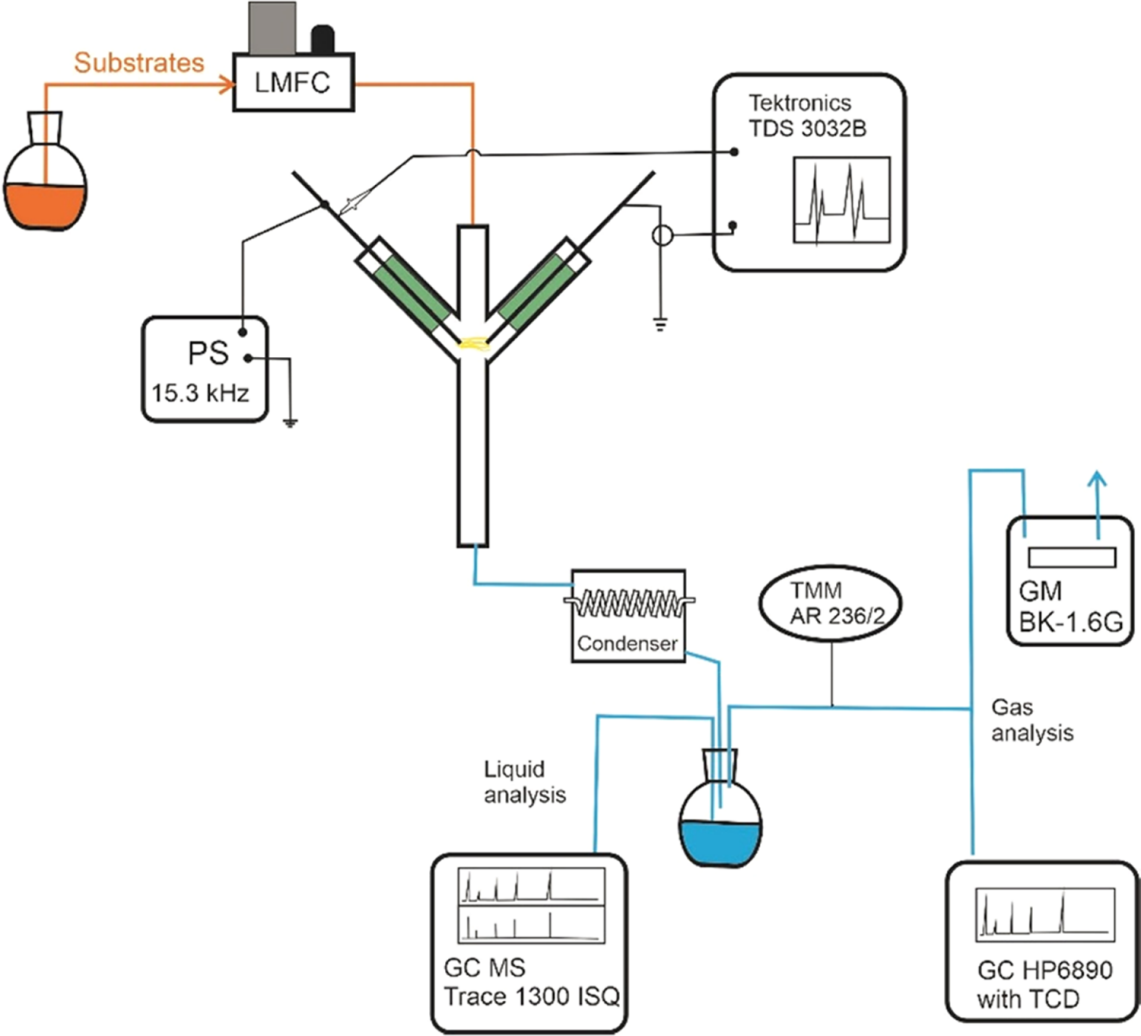
Apparatus diagram.

**Figure 2:**
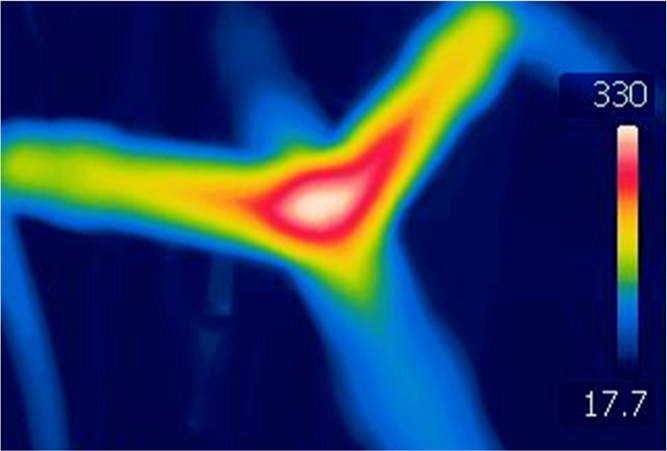
Reactor wall temperature.

The methodology and apparatus used in the research were similar to those used in our earlier studies described in our previous papers.^14,16−18^ A spark reactor powered by an alternating current power supply was used in the research. The apparatus diagram and the devices’ names are shown in Figure 1. The spark discharge was generated between two stainless steel electrodes with a diameter of 3.2 mm, spaced 6 mm apart. The reactor casing was made of quartz tubes with an internal diameter of 8 mm. The temperature of the reactor walls in the area of the plasma zone exceeded 300 °C (Figure 2). The peak voltage is about 0.9 kV. The maximum current is approximately 100 mA. The electron density ranged^19,20^ from 10^16^ to 10^18^ cm^–3^. The research was carried out with constant discharge power (25 W) and total feed flow (1 mol/h). During all of the measurements, a constant total feed flow was used to keep the residence time in the reactor and the specific energy per particle fed to the reactor constant. The water/ethanol molar ratio varied from 2 to 6. The temperature of the gases and the water vapor content were measured with an Apar meter with an AR236/2 sensor. Other components of the gas stream were measured with a Hewlett Packard gas chromatograph with a thermal conductivity detector. The condensate composition was measured with a Thermo Scientific gas chromatograph with a single quadrupole mass detector. The amount of coke was measured by gravimetric method.

The conversion of substrates, hydrogen yield, energy efficiency, and selectivity of ethanol conversion to carbon-containing products was calculated from the following formulas:
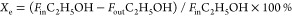
3
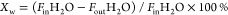
4
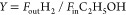
5
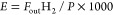
6
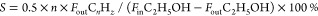
7where:

*X*_e_—ethanol conversion, %

*X*_w_—waterconversion, %

*S*—selectivity, %

*P*—discharge power, W

*Y*—hydrogen yield, mol(H_2_)/mol(C_2_H_5_OH)

*E*—energy efficiency, mol(H_2_)/kW h

*p*—products

*F*_in_C_2_H_5_OH—input ethanol flow, mol/h

*F*_out_C_2_H_5_OH—output ethanol flow, mol/h

*F*_in_H_2_O—input water flow, mol/h

*F*_out_H_2_O—output water flow, mol/h

*F*_out_H_2_—hydrogen flow, mol/h

*F*_out_C_*n*_H_*z*_—carbon-containing product flow, mol/h

*n*—number of carbon atoms in the product

*z*—number of hydrogen atoms in the product

## 3 Results and Discussion

**Figure 3:**
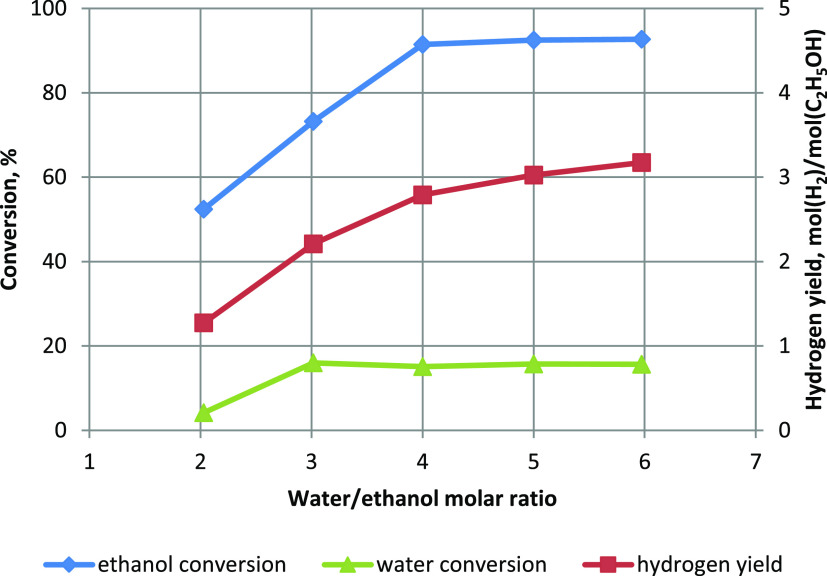
Influence of water content on conversion and hydrogen yield.

**Figure 4:**
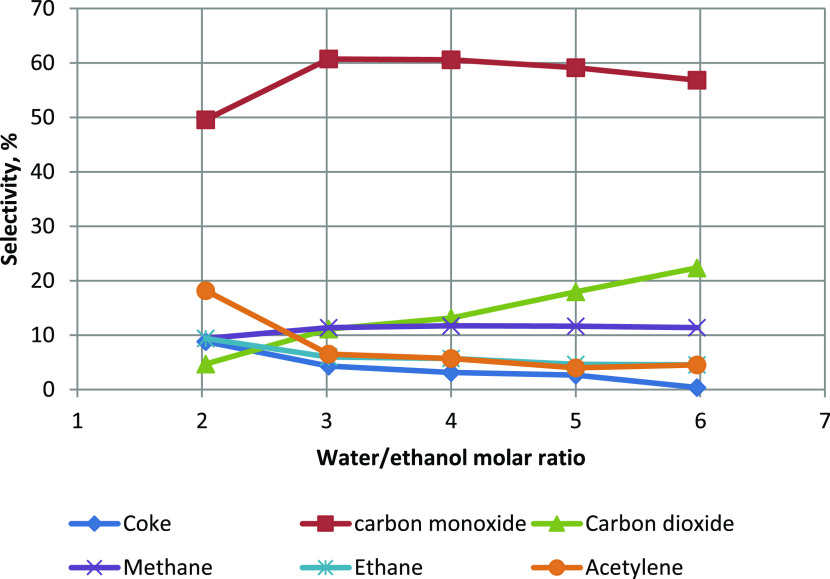
Influence of water content on the selectivity of ethanol conversion to carbon-containing products.

Figure 3 shows the substrate conversion and hydrogen yield. The ethanol conversion and hydrogen yield increased with increasing water/ethanol molar ratio. In contrast, the steam conversion decreased. Moreover, the water conversion was always several times lower than the ethanol conversion. It was expected when excess steam was used. However, even when the water/ethanol molar ratio was less than the stoichiometric ratio, the ethanol conversion was twice as high as the water conversion. It indicates that ethanol reacted more quickly than water. Computer simulations also showed that the dissociation rate constant of ethanol in collisions with electrons is many times greater than the dissociation rate constant of water.^21^ This is due to the presence of several chemical bonds in ethanol that are weaker than the chemical bonds in water.^14,22−25^ Therefore, several products can be formed from ethanol. In the dehydration reaction (eq 8), ethylene is formed, which, in subsequent reactions, produces acetylene (eq 9) and coke (eq 10).
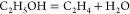
8
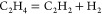
9
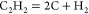
10Another direction of ethanol decomposition leads to the formation of methane
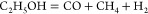
11These reactions competing with the reforming of ethanol (eq 12) lead to a reduction in the hydrogen yield. The hydrogen yield ranged from 1.2 to 3.2 mol(H_2_)/mol(C_2_H_5_OH) and increased with increasing water/ethanol molar ratio. If the process ran according to the desired reactions (eqs 12 and 13)
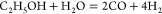
12
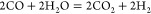
13six moles of H_2_ would be produced from one mole of ethanol. The excess of water favors these desired reactions, which was confirmed by the increase in the selectivity of ethanol-to-CO_2_ conversion (Figure 4) and the increase in the hydrogen yield (Figure 3). Additionally, a large amount of steam in the substrates inhibited the ethanol dehydration reaction (eq 8) and reduced the selectivity of ethanol conversion to ethylene, acetylene, and coke (Figure 4). The excess of water did not affect the selectivity of ethanol-to-methane conversion (eq 11) because, in this reaction, water is absent. A reduction of ethanol-to-methane conversion selectivity (eq 11) at a low water/ethanol molar ratio resulted from the acceleration of the dehydration reaction (eq 8). Increasing the importance of ethanol dehydration (eq 8) increased the selectivity of ethanol conversion to ethylene, acetylene, and coke. This is an expected effect since an increase in ethylene concentration promotes the formation of acetylene (eq 9), and, consequently, an increase in acetylene concentration promotes the formation of coke (eq 10). The selectivity of ethanol conversion to these three products (C_2_H_4_, C_2_H_2_, and coke) reached a total value of ∼30%. A high coke production (360 mg/h) is particularly disadvantageous since a filter should be frequently replaced.

The water/ethanol molar ratio of 2 resulted in high coke production and was the minimum value at which the reactor operated stably.

An attempt was made to reduce hydrogen production from the mixture with a water/ethanol molar ratio of 1. However, the amount of coke formed was so high that it was not entirely removed from the reactor but deposited on its walls. Within an hour, the accumulating coke led to short circuit of the electrodes. For a water/ethanol molar ratio of 2 or more, coke did not accumulate on the reactor walls, and the reactor did not require cleaning. A high water/ethanol molar ratio resulted in low coke production (12 mg/h). The change in the selectivity of ethanol-to-coke conversion supports the assumption that excess water prevents coke formation.

Additional advantages of using high water/ethanol molar ratios are high ethanol conversions (99%) and high hydrogen yields (3.2 mol(H_2_)/mol(C_2_H_5_OH)).

**Figure 5:**
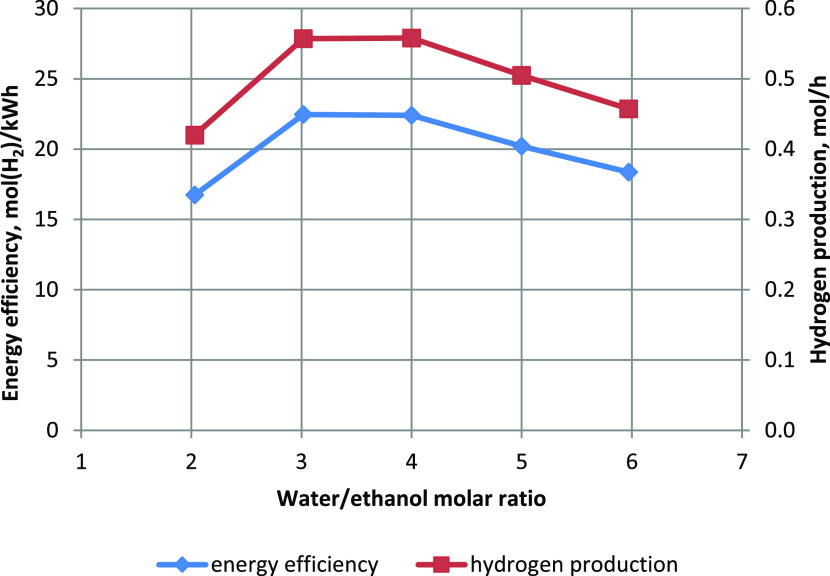
Influence of water content on energy efficiency and hydrogen production.

The water/ethanol molar ratio affected the energy efficiency of hydrogen production (Figure 5), which was ∼22.5 mol(H_2_)/kW h for water/ethanol molar ratios of 3 and 4. Hydrogen production was also the highest (Figure 5) for these conditions. Increasing the water/ethanol molar ratio to 5 and 6 resulted in a reduction in energy efficiency. The hydrogen production also decreased as the ethanol input stream decreased. Decreasing the water/ethanol molar ratio to 2 also reduced hydrogen production and energy efficiency. It was a surprising effect as the ethanol feed stream was the largest. However, under water shortage conditions, the ethanol dehydration reaction (eq 6) was thermodynamically favored, causing a decrease in hydrogen production. It also resulted in a reduction in energy efficiency. The energy efficiency of hydrogen production in spark discharge is similar to that achieved in microwave discharge^6^ and higher than in corona and barrier discharges,^10,12^ where the gas temperature is low.

**Figure 6:**
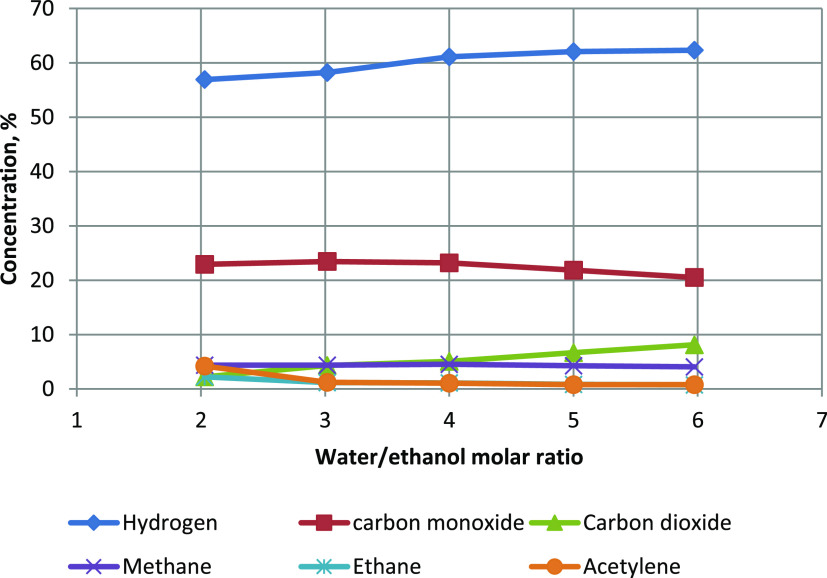
Influence of water content on gas composition.

The water content in the mixture introduced into the reactor significantly affected the composition of the gas mixture produced (Figure 6). Hydrogen and carbon dioxide concentrations increased with increasing water/ethanol molar ratio. On the other hand, carbon monoxide, ethylene, and acetylene concentrations decreased. The concentration of methane remained almost constant. The hydrogen concentration was high, ranging from 57 to 62%. The concentration of carbon monoxide was also high, ranging from 20 to 23%. The concentration of carbon dioxide was surprisingly low and ranged from 2 to 8%. Even when the water/ethanol molar ratio was 6, corresponding to a water/carbon ratio of 3, the CO_2_ concentration was low (8%), and the CO concentration remained high (20%). A higher CO concentration than CO_2_ is typical for plasma ethanol steam reforming.^6,7,10,12^

Since the use of a significant excess of water did not cause a considerable reduction in CO concentration and caused a reduction in the energy efficiency of the process, the optimal water/ethanol molar ratios are 3 and 4. It can be assumed that to increase the hydrogen yield, gases leaving the plasma reactor should be directed to the installation of CO conversion with steam, as it is during the production of hydrogen from natural gas. In steam reforming of natural gas, the CO concentration (19–12%) is also higher than the CO_2_ concentration (7–10%). In the subsequent stages of medium- and low-temperature water–gas shift reactions, the CO concentration drops to ∼0.3%, and the CO_2_ concentration increases to ∼20%.

Ethanol decomposition (eqs 8 and 11) and ethanol steam reforming (eq 12) were efficiently performed under plasma conditions. On the other hand, the water–gas shift reaction (eq 13) was inefficient. It is due to the energetic effects of the reactions. Reaction (eq 13) is an exothermic reaction, and under spark discharge conditions, endothermic reactions and high-temperature processes take place effectively.^28−34^ The reactions (eq 8), (eq 11), and (eq 12) are endothermic and therefore work well at the high temperatures that occur in spark discharges.

Coke and CO_2_ can also be formed in the Boudouard reaction (eq 14).
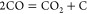
14

Excess water favors the formation of CO_2_ in the Bosch process (eqs 15 and 16), which is advantageous because it allows more hydrogen to be produced.
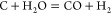
15
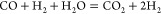
16The presence of coke may be the reason for hydrogen consumption toward the formation of hydrocarbons (eqs 17–20).
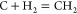
17
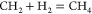
18
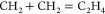
19
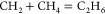
20Reactions with coke (eqs 15 and 17) require high temperatures. Therefore, they can occur in the plasma zone. In contrast, coke deposited on the filter is stable because the temperature outside the plasma zone quickly approaches ambient temperature (Figure 2), which is too low for coke to react.

Water content may increase ethanol conversion as water is a source of hydrogen and hydroxyl radicals (eq 21).
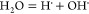
21Liu et al.^26^ reported that these radicals account for more than half of ethanol consumption. The study of plasma water splitting processes shows that the water conversion is low and the energy efficiency of the process is very low, up to 0.12 mol(H_2_)/kW h.^27^ This means that the hydroxyl and hydrogen radicals reproduce water if no other reactants exist.

## 4 Conclusions

Water content is essential for steam reforming of ethanol. The highest hydrogen production and energy efficiency were achieved when the water/ethanol molar ratio was stoichiometric or slightly higher than or equal to 3 or 4. As the coke production decreased rapidly and the ethanol conversion and hydrogen production efficiency increased with increasing water content, the optimal water/ethanol molar ratio was 4. With this ratio, the coke production, ethanol conversion, hydrogen yield, and energy efficiency were 132 g/h, 92%, 2.8 mol(H_2_)/mol(C_2_H_5_OH), and 22.5 mol(H_2_)/kW h, respectively. Increasing the water content increased the ethanol conversion and hydrogen yield. Additionally, the coke production decreased. However, the reduced energy efficiency was the negative effect of increasing the water content beyond the optimal values. For a water/ethanol molar ratio greater than 2, coke did not deposit on the reactor walls and did not interfere with its operation. The coke was removed from the reactor, along with the stream of produced gases.
